# Dexmedetomidine vs. propofol on arrhythmia in cardiac surgery: a meta-analysis of randomized controlled trials

**DOI:** 10.3389/fcvm.2024.1433841

**Published:** 2024-10-10

**Authors:** Juan Peng, Yifan Wu, Lin Li, Panpan Xia, Peng Yu, Jing Zhang, Xiao Liu

**Affiliations:** ^1^ Department of Anesthesiology, The Third Hospital of Nanchang, Nanchang, Jiangxi, China; ^2^ Department of Anesthesiology, The Second Affiliated Hospital of Nanchang University, Nanchang, Jiangxi, China; ^3^ Department of Traditional Chinese Medicine, Fujian University of Traditional Chinese Medicine, Fuzhou, China; ^4^ Department of Endocrinology and Metabolism, The Second Affiliated Hospital of Nanchang University, Nanchang, China; ^5^ Department of Cardiology, Sun Yat-sen Memorial Hospital of Sun Yat-sen University, Guangzhou, Guangdong, China; ^6^ Guangdong Provincial Key Laboratory of Arrhythmia and Electrophysiology, Guangdong Provincial Key Laboratory of Malignant Tumor Epigenetics and Gene Regulation, Guangdong-Hong Kong Joint Laboratory for RNA Medicine, Sun Yat-Sen University, Guangzhou, Guangdong, China

**Keywords:** cardiac surgery, dexmedetomidine, meta-analysis, propofol, ventricular arrhythmias

## Abstract

**Background:**

Dexmedetomidine (DEX) and propofol are popular anesthetics, but it remains unknown whether DEX reduces the incidence of arrhythmias compared with propofol after cardiac surgery.

**Methods:**

We performed a comprehensive search for RCTs (Randomized Controlled Trials) that compared the incidence of arrhythmias between DEX and propofol in adults who had undergone cardiac surgery across three databases (PubMed, Embase, the Cochrane Library), and ClinicalTrials.gov up to October 3, 2023. The primary outcome was ventricular arrhythmias, the secondary outcomes were bradycardia and atrial fibrillation (AF).

**Results:**

Our analysis included 7 RCTs with 1,004 patients (mean age: 64.37, male: 71.11%) undergoing cardiac surgery, and the incidence of in-hospital arrhythmia was 22.01% (ventricular arrhythmias 2.75%, bradycardia 3.33%, AF 18.63%). Perioperative or postoperative use of DEX reduced the incidence of in-hospital ventricular arrhythmias [Odds Ratio (OR) 0.14, 95% Confidence Interval (CI) 0.03–0.66], but increased the risk of in-hospital bradycardia (OR 2.88, 95% CI 1.02–8.17) compared with propofol. The trial sequence analysis verified the adequacy of sample size and robustness of the ventricular arrhythmias and bradycardia. There was no significant reduced incidence of the use of DEX in the incidence of AF (OR 0.69, 95% CI 0.36–1.29). The GRADE assessment indicated a high certainty for ventricular arrhythmias and bradycardia and a moderate certainty for AF.

**Conclusions:**

Our findings suggested the use of DEX reduces in-hospital ventricular arrhythmias but increases bradycardia incidence compared to propofol in adult patients undergoing cardiac surgery. Further studies are needed to assess the impact of dexmedetomidine on atrial fibrillation compared to propofol.

**Systematic Review Registration:**

http://www.crd.york.ac.uk/prospero/ PROSPERO, identifier (CRD42023482193).

## Graphical Abstract

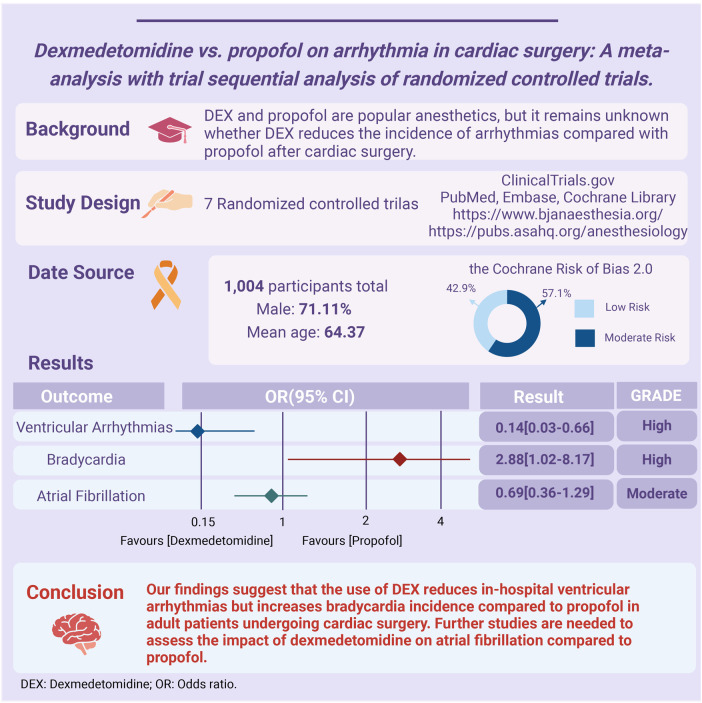

## Introduction

Complications after cardiac surgery remain a problem, with an incidence ranging from 14.4% to 30.1% (1). Among these complications, arrhythmias are noteworthy. Common risk factors that predispose individuals to ventricular arrhythmias following cardiac surgery include hemodynamic instability, hypoxia, hypovolemia, coronary artery disease, infarction, and reperfusion injury, among others (2).

Dexmedetomidine (DEX) is a widely used highly selective alpha-2 receptor agonist in the perioperative phase and offers anxiolytic, sedative, and moderate analgesic effects while minimizing respiratory depression. In cardiac surgery, DEX finds utility in both the induction and maintenance of anesthesia as well as in hemodynamic management. Known for its minimal impact on ventilation and its ability to maintain arousal effectively (3). DEX can effectively reduce sympathetic activity, sinus node excitability, and the patient's heart rate (4). Propofol is another a prevalent intravenous anesthetic. It also has extensive application in anesthesia induction and maintenance, as well as for prolonged sedation in intensive care units (5, 6).

Due to their favorable pharmacokinetics and ability to induce rapid and reversible sedation, both DEX and propofol have emerged as the primary choices for anesthesia and intensive care worldwide (7). While previous meta-analyses (8–11) have primarily focused on comparing DEX to placebos concerning arrhythmias, a randomized controlled trial (3) has demonstrated DEX's effectiveness in reducing arrhythmias in post-cardiac surgery patients. However, there is a notable knowledge gap as no studies have directly compared the effects of DEX and propofol on arrhythmias in patients undergoing cardiac surgery. Given this background, we conducted a comprehensive meta-analysis of clinical randomized trials to compare the efficacy of DEX and propofol on arrhythmias in patients undergoing cardiac surgery.

## Methods

### Protocol registration and search strategy

The protocol was registered with PROSPERO (International Prospective Register of Systematic Reviews. https://www.crd.york.ac.uk/PROSPERO/ -registration number-CRD42023482193), and the results were reported following the Preferred Reporting Items for Systematic Reviews and Meta-Analyses (PRISMA) checklist (12) (Supplementary Table S1). Two authors (X.L. and Y.F. W.), independently conducted the database search, selection, data extraction, and statistical analysis. We performed a comprehensive search across electronic databases (PubMed, EMBASE, and Cochrane Library), ClinicalTrails.gov, website of The Royal College of Anaesthetists (https://www.bjanaesthesia.org) and American Society of Anesthesiologists (https://pubs.asahq.org/anesthesiology). Additionally, we searched related literature using Google Scholar. The search encompassed studies published up until October 3, 2023. No language restrictions were applied during the search process. To ensure comprehensive coverage of relevant literature, we utilized a combination of MeSH terms and keywords in our search methodology. The search terms we employed were as follows: (“Dexmedetomidine” OR “Precedex” OR “Dexmedetomidine Hydrochloride”) AND (“Propofol” OR “2,6-Diisopropylphenol” OR “2,6-Bis [1-methylethyl]phenol”) AND (“Cardiac Surgery” OR “Heart Surgical Procedure”) AND (“Arrythmia” OR “Cardiac Dysrhythmia” OR “Atrial Fibrillation” OR “Ventricular Fibrillation” OR “Atrial Flutter” OR “Ventricular Tachycardia” OR “Atrial Tachycardia”) (Supplementary Table S2).

### Selection criteria and study selection

For this meta-analysis, we applied the PICOS criteria as follows: (1) Participants: Adults undergoing cardiac surgery; (2) Intervention: Use of dexmedetomidine (3) Comparison: Use of propofol; (3) Outcomes: Primary outcome: Ventricular arrhythmias; Secondary outcome: Bradycardia and atrial fibrillation (AF); (4) Study Design: Randomized controlled trials (RCTs). We excluded systematic reviews, observational studies, reviews, animal studies, studies not comparing DEX and propofol, and studies involving children or newborns.

Ventricular arrhythmias include premature ventricular contractions, ventricular tachycardia, ventricular flutter, and ventricular fibrillation. Ventricular tachycardia is defined as arrhythmias with a frequency greater than 100 beats per minute of three or more consecutive complex waves originating in the ventricles. Bradycardia refers to a heart rate slower than 60 beats per minute. AF is a common arrhythmia with rapid, irregular atrial beating, detectable on an ECG as an irregular rhythm without distinct *P* waves.

We used EndNote X9.1 software (Thomson Reuters, New York, NY) to manage search results, including the automated removal of duplicate citations followed by a manual review to ensure accuracy. We then screened the remaining titles and abstracts to identify reports aligning with our inclusion criteria. Any uncertainties were resolved through discussion and consensus. Finally, we obtained full reports of the citations likely to meet the criteria, ensuring a systematic and rigorous selection of relevant studies.

### Data collection and quality assessment

We extracted the following data from the included studies: (1) first author name, (2) publication year, (3) Country, (4) Source of participants, (5) study design, (6) mean age, (7) overall population and the number of cases, (8) Surgery type, (9) DEX and Propofol dose, time and duration, (10) Outcome, (11) Odds ratios [ORs] and the corresponding 95% confidence intervals [CIs]. Data were inspected independently by three authors (L.X. YF.W. and YZ.H.), and conflicts were resolved by consensus after a detailed discussion.

Quality was used by the Cochrane Risk of Bias 2.0(RoB2) tool for RCTs. Its assessment focuses on five key domains: the randomization process, deviations from intended interventions, missing outcome data, measurement of the outcome, and selection of the reported result. In each of these domains, we assigned a risk rating of “low”, “some concerns”, or “high”, The overall risk of bias for each trial was determined based on the highest risk observed in any single domain.

### Statistical analysis

We employed a random-effects model for meta-analysis. To evaluate the true variation among the included studies, we utilized the Cochran *Q*-test and Tau^2^. Statistically significant heterogeneity was considered when the *P*-value was less than 0.10 for the *Q* statistic. I^2^ were used to assess the study-level inconsistencies, prompting researchers to explore potential sources of variation among studies.

We graded the certainty of the evidence of pooled results based on the GRADE (Grading of Recommendations Assessment, Development, and Evaluation) framework (13). Any discrepancies between the two reviewers when evaluating each domain for the selected outcomes were resolved through consensus discussions. To streamline the assessment and presentation of the evidence, we utilized the GRADEpro GTD tool (https://gdt.gradepro.org/app/) and generated evidence profile tables. Quality of evidence includes three grades, high-certainty evidence, moderate-certainty evidence, low-certainty evidence and very low-certainty evidence. We performed trial sequential analyses using TSA (trial sequence analysis) v0.9.5.10, where we employed a significance level (α) set at 0.05 and a statistical power of 80%.

Pre-plan subgroup analyses were performed, including types of ventricular tachycardia, age, sex, dose of DEX, region and type of surgery. To assess potential publication bias, we employed funnel plots, Egger's test, and Begg's test. We also performed a sensitivity analysis by excluding each study one at a time to assess the robustness of the results. Data analysis was employed by RevMan software, version 5.4.1 (Nordic Cochrane Center, Copenhagen, Denmark). All statistical tests were conducted as double-sided tests, with *P* < 0.05 was considered as statistical significance.

## Results

### Literature search

**Figure 1:**
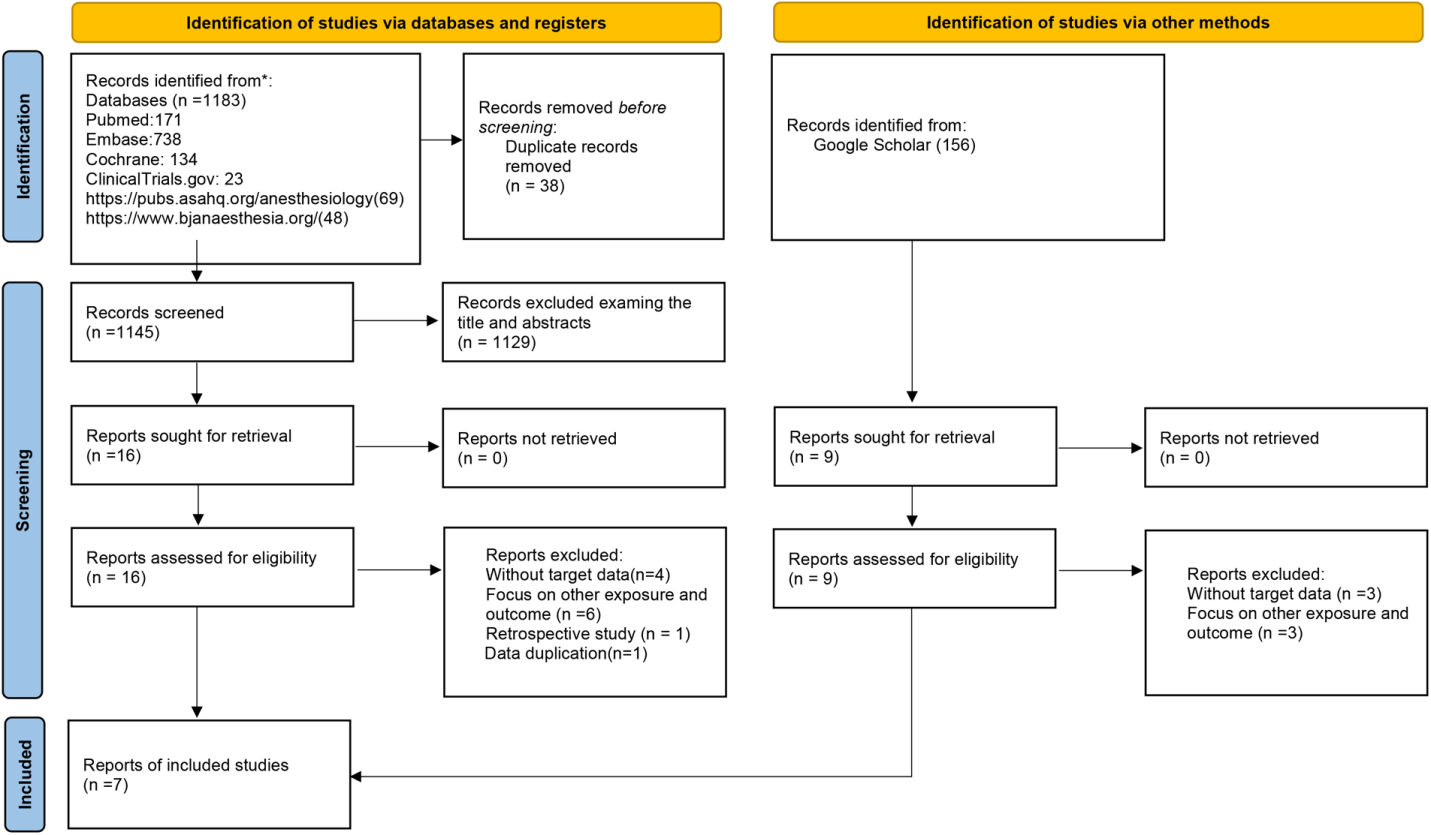
Flow chart of the study selection process. Seven randomized controlled trials (RCTs) studies were included by searching the PubMed, EMBASE, and Cochrane Library database and ClinicalTrails.gov.

The study search process is detailed in Figure 1. Initially, 1,183 studies were identified [PubMed: 171; Embase: 738; Cochrane Library: 134; ClinicalTrials.gov: 23; website of The Royal College of Anaesthetists (https://www.bjanaesthesia.org/): 69; American Society of Anesthesiologists (https://pubs.asahq.org/anesthesiology): 48]. Additionally, we retrieved grey literatures from Google Scholar yielding 156 additional studies. After removing duplicate records, 38 studies were excluded. Following a thorough screening of titles and abstracts, 1,276 studies unrelated to the subject were excluded. Subsequently, we reviewed 25 full texts. Among these articles, 18 were excluded for the following reasons: (1) lack of data on the incidence of arrhythmia (*n* = 7); (2) focus on other intervention and outcome (*n* = 9); (3) observational study (*n* = 1); (4) data duplication (*n* = 1). Ultimately, 7 RCTs met the inclusion criteria (14–20). All excluded studies along with the reasons for exclusion (*n* = 18) are presented in Supplementary Table S3.

### Study characteristics and study quality

Table 1 provides a comprehensive overview of 7 RCTs studies spanning the years 2003–2020, encompassing a total of 1,004 patients. Notably, the sample sizes ranged from 64 to 295 individuals, while the average age of study participants ranged from 54.8 to 72.6 years. All 7 RCTs reported outcomes related to AF (14–20), while four studies reported on bradycardia (14, 15, 17, 20), and three focused on ventricular arrhythmias (18–20). Diagnosis of arrhythmic events was detected by electrocardiogram (ECG), including ventricular arrhythmias, bradycardia, and AF, and follow-up ranged from one day to the end of ICU hospitalization. Five studies involved coronary artery bypass surgery (14, 17–20), 2 of which utilized non-cardiopulmonary bypass coronary artery grafting (OPCABG) (14, 18), while 3 studies employed coronary artery bypass graft (CABG) (17, 19, 20). Two studies (19, 20) started continuous infusion of DEX intraoperatively, and 5 studies (14–18) started continuous infusion of DEX postoperatively. Geographically, 3 of the seven studies originated from Asian countries (14, 15, 18), 3 from North American countries (16, 19, 20), and 1 from a European (17). In terms of quality assessment, 3 were classified as low risk (14–16), while the remaining four were categorized as medium risk (17–20), 2 of these studies (18, 20) had opaque randomization, 2 studies (15, 17), health care providers or people administering the intervention may have been aware of the intervention or downgraded if they did not provide hidden information about the intervention (Supplementary Figure S1).

**Table 1:** Characteristics of included randomized controlled trials in the meta-analysis of dexmedetomidine vs. propofol effect on arrhythmia in patients undergoing cardiac surgery.

Author (Publication year), Country	Patients	Mean age, sex ratio, sample size	Surgery type	DEX intervention, intervention time	Propofol infusion	Outcomes and follow up	OR (95% CI)
Zi 2020, China	Patients undergoing OPCABG surgery	65.4, 2.08, 123	OPCABG	0.2–1.0 μg/kg/h, mechanical ventilation after analepsia	0.1–3 mg/kg/h	AF 5 days	0.39 (0.17–0.93)
						Bradycardia 5 days	3.05 (0.31–30.17)
George 2016, Canada	Undergoing elective complex cardiac surgery	72.7, 3.07, 183	On-pump cardiac surgery	0.4 μg/kg bolus followed by 0.2–0.7 μg/kg/h, ICU admission-extubation	25–50 μg/kg/min	AF 5 days	1.28 (0.71–2.29)
Liu 2016, China	Adults, elective cardiac surgery with CPB, admitted to ICU	62.5, 0.66, 88	On-pump cardiac surgery	0.2–1.5 μg/kg/h, ICU admission- extubation	0.3–3 mg/kg/h	AF During ICU	0.28 (0.10–1.30)
						Bradycardia During ICU	4.30 (0.46–40.12)
Karaman 2015, Turkey	Patients who had elective CABG surgery	62.5, 6.11, 64	CABG	0.2–1.0 μg/kg/h, ICU admission-extubation	1.0–3.0 mg/kg/h	AF During ICU	2.21 (0.19–25.64)
						Bradycardia During ICU	2.21 (0.19–25.64)
Ren 2013, China	Patients who were undergoing OPCAB surgery	60, 2.68, 162	OPCAB	0.2–0.5 μg/kg/h, following the first vascular anastomosis grafting∼12 h in the ICU	2–4 mg/kg/h	Ventricular arrhythmias 3 days	0.16 (0.02–1.33)
						AF 3 days	0.19 (0.02–1.66)
Corbett 2005, US	All potential nonemergent CABG patients requiring postoperative ICU sedation during MV	63, 4.56, 89	CABG	1 μg/kg induction then maintained by 0.4 μg/kg/h, during bypass surgery −1 h post-extubation	0.2–0.7 μg/kg/h	VF During ICU	0.35 (0.01–8.79)
						AF During ICU	3.28 (0.13–82.77)
Herr 2003, US and Canada	Adults undergoing CABG surgery	61.9, 13.75, 295	CABG	1.0 μg/kg induction then maintained by 0.2–0.7 μg/kg/h, during sternal closure-24 h in the ICU	No detailed data	Ventricular arrhythmias During ICU	0.06 (0.00–1.11)
						AF During ICU	0.99 (0.43–2.29)
						Bradycardia During ICU	2.53 (0.48–13.28)

DEX, dexmedetomidine; AF, atrial fibrillation; VF, ventricular fibrillation; CABG, coronary artery bypass graft; OPCAB, off-pump coronary artery bypass; OPCABG, off-pump coronary artery bypass graft; CPB, cardiopulmonary bypass; ICU, intensive care unit.

### Primary outcome

#### Ventricular arrhythmias

**Figure 2:**
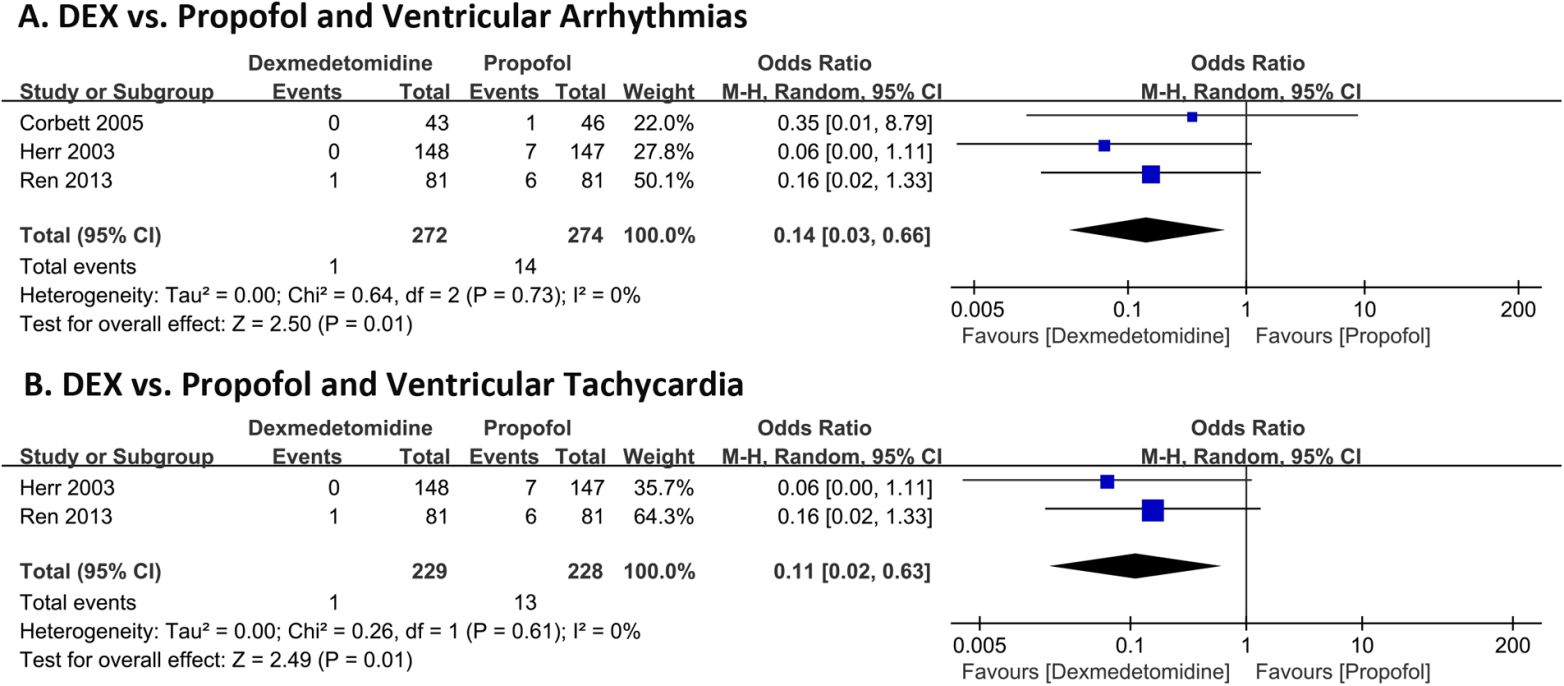
Forest plot of the risk of ventricular arrhythmias of dexmedetomidine compared with propofol in patients after undergoing heart surgery. Ventricular arrhythmias **(A)** and ventricular tachycardia **(B)**.

Figure 2A shows the incidence of in-hospital ventricular arrhythmias outcomes of DEX compared with propofol in patients undergoing cardiac surgery. Three RCTs with 546 patients were included. The outcome of 2 RCTs (18, 20) was ventricular tachycardia and 1 RCT (19) was ventricular fibrillation. A total of 15 (2.74%) people experienced ventricular arrhythmias, of which 1 (0.37%) developed ventricular arrhythmia outcomes in the DEX group and 14 (5.11%) in the propofol group. The pooled results revealed patients administered with DEX exhibited a significantly reduced risk of ventricular arrhythmias (OR 0.14, 95% CI 0.03–0.66, *P* = 0.01, *Q*-test *P* = 0.73, Tau^2^ = 0.00, I^2^ = 0%). Subgroup analysis showed DEX significantly reduced the risk of ventricular tachycardia (VT) (OR 0.11, 95% CI 0.02–0.63, *P* = 0.01, *Q*-test *P* = 0.61, Tau^2^ = 0.00, I^2^ = 0%) Figure 2B.

### Second outcomes

#### Bradycardia

**Figure 3:**
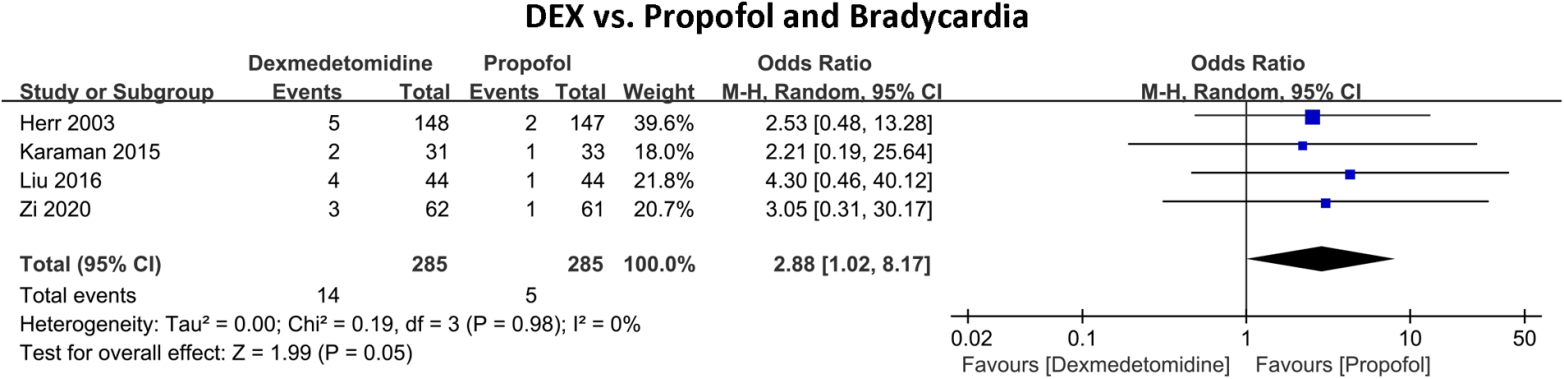
Forest plot of the risk of bradycardia of dexmedetomidine compared with propofol in patients after heart surgery.

Figure 3 shows the incidence of in-hospital bradycardia outcomes of DEX compared with propofol in patients undergoing cardiac surgery. Four RCTs with 570 patients and 19 bradycardia events were included. 14 (4.91%) patients developed bradycardia in the DEX group and 5 (1.75%) in the propofol group. The pooled results revealed patients who received DEX exhibited a notably increased risk of bradycardia (OR 2.88, 95% CI 1.02–8.17, *P* = 0.05, *Q*-test *P* = 0.98, Tau^2^ = 0.00, I^2^ = 0%).

#### Atrial fibrillation

**Figure 4:**
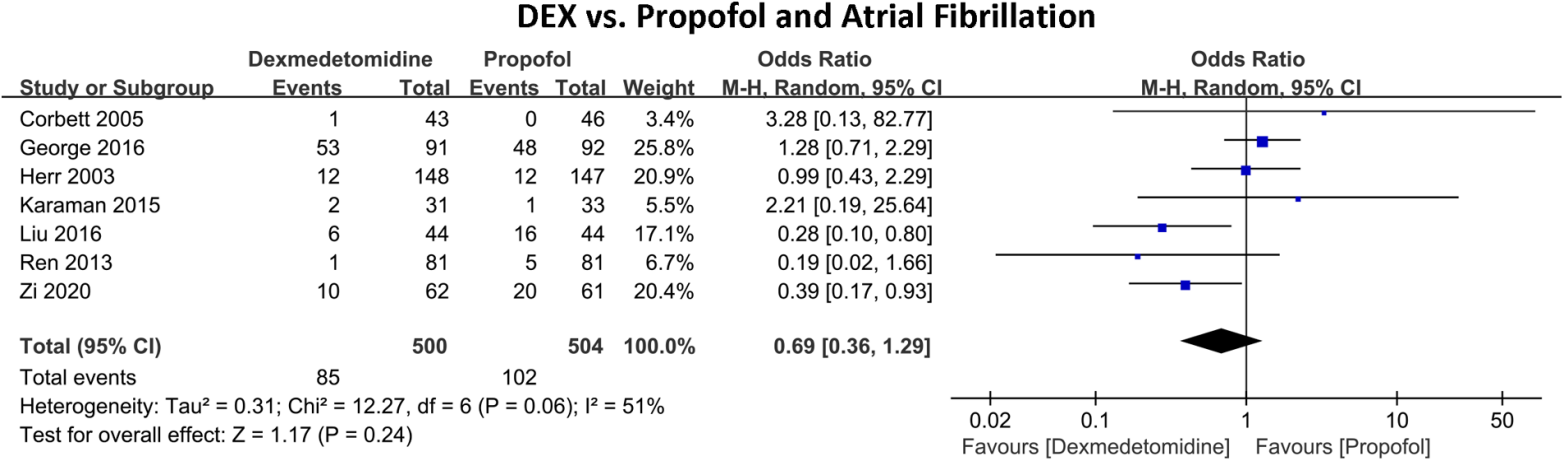
Forest plot of the risk of atrial fibrillation of dexmedetomidine compared with propofol in patients undergoing heart surgery.

Figure 4 shows the incidence of in-hospital AF between DEX and propofol. Seven RCTs with 1,004 patients were included. A total of 187 (18.63%) people experienced AF, of which 85 (17.00%) developed AF in the DEX group and 121 (20.24%) in the propofol group. The pooled results indicated that in comparison to propofol, DEX did not exhibit a significant reduction in the risk of AF (OR 0.69, 95% CI 0.36–1.29, *P* = 0.24, *Q*-test *P* = 0.06, Tau^2^ = 0.31, I^2^ = 51%).

#### Sensitivity analysis, subgroup analysis, and publication bias

No significant subgroup differences were observed in stratified analyses based on region and type of surgery, as detailed in Table 2. Additionally, funnel plot assessment, Egger's test, and Begg's test, did not reveal any significant indications of potential bias, Supplementary Figure S2. Furthermore, sensitivity analysis by omission of each individual study demonstrated the stability of the results Supplementary Figure S3.

**Table 2:** Subgroup analyses included randomized controlled trials in the meta-analysis of dexmedetomidine vs. Propofol effect on arrhythmia in patients undergoing cardiac surgery.

Items		Number of trials	OR (95% CI)	*P*	*P* *_h_	*P* **
1. Bradycardia						
Result of primary analysis		4	2.88 [1.02, 8.17]	0.05	0	-
Asian	Yes	2	3.64 [0.73, 18.01]	0.11	0	-
	No	2	2.43 [0.62, 9.58]	0.21	0	0.71
CABG	Yes	2	3.09 [1.27–7.52]	0.01	0	–
	No	2	3.64 [0.74–17.99]	0.11	0	0.86
2. Ventricular arrhythmias						
Result of primary analysis		3	0.14 [0.03–0.66]	0.01	0	–
Asian	Yes	1	0.16 [0.02, 1.33]	0.09	0	–
	No	2	0.13 [0.02, 1.15]	0.07	0	0.42
CABG	Yes	2	0.13 [0.02, 1.15]	0.07	0	–
	No	1	0.16 [0.02, 1.33]	0.09	0	0.42
3. Atrial fibrillation						
Result of primary analysis		7	0.69 [0.36–1.29]	0.24	51	–
Asian	Yes	3	0.33 [0.17, 0.61]	<0.01	0	–
	No	4	1.20 [0.71, 2.03]	0.51	0	<0.01
CABG	Yes	3	1.15 [0.53, 2.47]	0.73	0	–
	No	4	0.48 [0.21, 1.13]	0.09	62	0.14

CABG, coronary artery bypass graft.

* *P* for within-group heterogeneity.

** *P* for subgroup difference.

#### GRADE assessments and trial sequential analysis

The Grade framework assessment resulted in a downgrade due to moderate heterogeneity observed in the pooled results for AF. Consequently, the GRADE assessment indicates a high level of quality in the incidence of ventricular arrhythmias and bradycardia, while it indicates a moderate level of quality in the incidence of AF (Supplementary Table S4).

The trial sequence analysis revealed a significant crossing of the cumulative Z-curve over the failure boundary, providing robust evidence that DEX was linked to an elevated risk of bradycardia and a diminished risk of ventricular arrhythmias compared to propofol. In contrast, the cumulative Z-curve did not cross the failure boundary, indicating insufficient evidence to conclude that DEX was associated with a reduced risk of AF compared to propofol. The alignment between trial sequential analysis and the primary meta-analysis enhances the credibility and reliability of our findings (Supplementary Figure S4).

## Discussion

### Major findings

In this meta-analysis, DEX reduced the risk of ventricular arrhythmias and increased the risk of bradycardia in patients after cardiac surgery compared to propofol. Our findings firstly indicated that DEX substantially reduces the risk of ventricular arrhythmias by 86% and the incidence of VT by 89% (*P* = 0.01), however, DEX increased the risk of bradycardia by 188% (*P* = 0.05). Conversely, there was no significant reduction in the risk of AF with DEX compared to propofol (OR = 0.69, *P* = 0.24). It is important to note that the sample size of patients is small, more RCTs are needed to confirm our results. In clinical practice, for high-risk populations prone to ventricular arrhythmias, such as patients with coronary artery disease, heart failure, dilated cardiomyopathy, or those who have undergone cardiac surgery, DEX may offer better outcomes compared to propofol by reducing the risk of ventricular arrhythmias and improving prognosis. Conversely, for individuals at high risk for bradycardia, including the elderly, patients with sinus node dysfunction, atrioventricular block, or those with cardiomyopathies and valvular heart diseases that affect cardiac conduction, propofol may potentially provide better prognostic benefits than DEX.

### Comparison with previous studies

Postoperative arrhythmias frequently occur and is associated with poor prognosis in patients undergoing cardiac surgery. According to previous reports, the incidence was around 3% for postoperative ventricular arrhythmias (21) 5% for bradycardia (22), 35% for AF (23, 24). Notably, the incidence of these conditions reported in this study (ventricular arrhythmias 2.75%, bradycardia 3.33%, AF 18.63%) differs from the averages found in existing literatures. Numerous reports and our previous study have discovered multiple risk factors for the post-operative arrhythmia (25, 26). Intervention for prevention of post-operative arrhythmia is still an appealing topic. In our study, we made a noteworthy discovery: the utilization of DEX postoperatively exhibited a robust reduction in the risk of ventricular arrhythmias (OR 0.14, *P* = 0.01) while concurrently elevating the risk of bradycardia (OR 2.88, *P* = 0.05) in comparison to the use of propofol. These findings are consistent with previous findings about the potential anti-arrhythmia effect of DEX in patients after cardiac surgery (11, 27, 28). For instance, Ling et al. (11) conducted a comprehensive meta-analysis of nine RCTs, elucidating that DEX exerts a potent anti-ventricular arrhythmic effect compared to other sedative agents administered post-cardiac surgery, such as propofol, morphine, and even a placebo (OR 0.24, 95% CI 0.09–0.64). Similarly, Zhong et al. (29), after analyzing data from six RCTs, reported a substantial decrease in the overall incidence of ventricular arrhythmias (RR 0.35, 95% CI 0.16–0.76), along with a significant reduction in the risk of tachycardia (RR 0.25, 95% CI 0.08–0.80) among patients undergoing cardiac surgery. Moreover, their study demonstrated a favorable safety profile for DEX when compared to a placebo (RR 2.78, 95% CI 2.00–3.87). In an early retrospective cohort study by Ji et al. (27), perioperative DEX use was linked to a decreased risk of arrhythmias post-coronary artery bypass grafting. Similarly, another retrospective cohort study found that continued use of DEX postoperatively significantly lowered the risk of borderline ectopic tachycardia in children with congenital heart disease undergoing CPB (30). Adding to this body of evidence, Chrysostomou et al. (31) conducted a prospective cohort study, specifically in pediatric patients after cardiothoracic surgery, and noted a marked reduction in the incidence of ventricular and supraventricular tachyarrhythmias with the perioperative use of DEX. In our research, despite a comprehensive evaluation of the analgesic treatment efficacy, we observed significant variability in the implementation of the analgesic protocols. For example, there were notable differences in the timing, duration of infusion, and actual dosage of DEX administered. These discrepancies could potentially impact the study outcomes, especially in evaluating the safety and efficacy of the treatment, and might introduce bias. Moreover, the outcomes we collected were not predefined primary endpoints, and conclusions drawn from secondary outcomes may lack reliability. Although the results for ventricular arrhythmias showed no heterogeneity, this does not imply absolute consistency or accuracy. Other factors such as patients' underlying medical conditions, concomitant medications, and differences in study designs could still influence the final conclusions. In patients with multiple concurrent cardiac conditions, a single intervention with DEX may not achieve optimal efficacy, and combination therapies could lead to clinical heterogeneity in the outcomes related to ventricular arrhythmias. Liu et al.'s meta-analysis (10) indicated that, although the optimal dose of DEX for preventing postoperative arrhythmias remains unclear, subgroup analyses showed that continuous infusion of DEX without a loading dose is both safe and effective, avoiding adverse hemodynamic effects. Additionally, the timing of DEX administration is crucial: preoperative or intraoperative administration appears to yield better efficacy and outcomes compared to postoperative administration. Therefore, even with low heterogeneity in the study, we must interpret the results with caution and avoid overlooking other potential confounding factors.

Dexmedetomidine for reduction of atrial fibrillation and delirium after cardiac surgery (DECADE) study previously showed DEX is not effective at reducing AF after cardiac surgery compared with placebo with high-quality evidence (3). Consistently, our research findings showed that the use of DEX does not significantly reduce the risk of AF when compared to propofol after cardiac surgery. Another RCT by Turan et al. demonstrated that initiating DEX infusion at the time of anesthesia induction did not reduce the occurrence of postoperative AF in patients undergoing cardiac surgery. However, it contradicts earlier studies, Peng et al. (8) conducted a meta-analysis comprising 15 RCTs, revealing that DEX usage notably decreased the risk of postoperative AF (POAF) following cardiac surgery compared to mixed of propofol, morphine, placebo, and other pharmaceuticals. Similarly, both meta-analysis of RCTs by Wang et al. (32) and Liu et al. (10) showed that perioperative administration of DEX significantly lowered AF incidence post-cardiac surgery compared to placebo and alternative anesthetics. We would like to explain this inconsistency with caution. Notably, no previous meta-analysis systematically assessed a directed comparison between propofol, the combined of mixed comparators (such as propofol, morphine) may increase the positive fault rate and make the results hard to be explained in clinical practice. Second, none of the 7 studies delineated specific exclusion criteria for patients with AF, and did not distinguish between new-onset and recurrent AF. Such inconsistencies might indirectly impact the reported AF cases and potentially overestimate the incidence of AF. Significantly, heterogeneity notably decreased upon excluding the study of patients with AF at baseline (16) (*P* = 0.08, I^2^ = 33%), rendering OR more significant (OR, 0.55; 95% CI 0.28–1.07). Furthermore, differences in control groups may contribute to result disparities. Consequently, the efficacy of DEX vs. propofol in mitigating AF incidence call for further study.

The incidence of POAF may also be influenced by the type of surgery, as evidenced by Gillinov et al.'s study (33), which reported a higher POAF occurrence in patients undergoing combined surgery or isolated valve procedures compared to those undergoing coronary artery bypass grafting (CABG) alone. However, subgroup analysis for CABG surgery did not yield statistically significant results, potentially due to the study's limited sample size. Furthermore, the efficacy of dexmedetomidine (DEX) in reducing POAF incidence compared to propofol may be dose-dependent. Wang et al.'s meta-analysis demonstrated a significant reduction in POAF incidence in the high-dose DEX group (1.5 μg·kg^−1^·h^−1^) compared to the low-dose DEX group (0.7 μg·kg^−1^·h^−1^) (18.93% vs. 25.54%). This finding indirectly suggests a dose-response relationship between DEX dosage and POAF risk reduction. However, potential heterogeneity may arise from variations in DEX infusion doses and timing, necessitating further validation through additional RCTs. Additional research is warranted to confirm these findings and elucidate optimal DEX dosage and administration protocols for POAF prevention in cardiac surgery patients.

Ethnicity may also be a factor influencing AF outcomes. Subgroup analyses in the meta-analysis conducted by Peng et al. (8) showed that DEX was effective in reducing the incidence of POAF in Asian populations, while the results of non-Asian studies were less consistent. This difference may be attributed to potential genetic, physiological, or environmental differences between ethnic groups. For example, previous studies have shown that Caucasian patients have a higher risk of developing POAF after cardiac surgery compared to patients from other ethnic backgrounds (34, 35). In addition, demographic factors such as age and gender also play a crucial role in the risk of developing AF. This may be due to differences in baseline risk and response to medications in these populations.

Propofol has been shown to prolong atrioventricular nodal conduction and indirectly improve AF (36, 37). Therefore, while there is a theoretical inverse association between DEX and the risk of AF (3), our study did not find a statistically significant difference in reducing AF when compared to propofol. The cumulative Z-curve in the sequential analysis of the trial did not cross the invalid boundary, which may be due to false negatives caused by insufficient sample size. This highlights the need for further randomized clinical studies to confirm and expand upon our findings.

### Potential mechanism

The preventive effect of DEX on arrhythmias may be related to the autonomic nervous system, inhibition of inflammatory response, and oxidative stress response (5, 38). Tachyarrhythmias, such as rapid heartbeats, can detrimentally affect cardiac function by reducing diastolic filling time and cardiac output, potentially leading to myocardial ischemia and hypotension. DEX, by activating G protein-coupled transmembrane *α*-2 receptors in the brain, posits a theoretical protective effect against myocardial ischemia (27, 39). This protection stems from its influence on the regulation of sympathetic activity, shifting the balance from the central to the peripheral nervous system, and enhancing cardiac function by elevating cAMP levels. Furthermore, it augments adenosine-induced coronary vasodilation, thereby contributing to myocardial protection. An additional mechanism underlying DEX's anti-arrhythmic properties involves its impact on vagus nerve activity (40). By modulating the release of inflammatory mediators and dampening the production of cytokines, it effectively curbs the cascade of inflammatory processes, ultimately reducing the extent of cardiac degeneration (41–43). The antioxidant capacity of DEX plays a crucial role in maintaining the health of cardiac tissues and may aid in reducing the likelihood of arrhythmias (44). DEX downregulates If currents through receptors other than α2-adrenergic receptors (α2-AR), resulting in a negative timing effect on sinus node function and inducing bradycardia (45, 46). However, blocking adrenergic β1 and α1 receptors is one of the pharmacologic actions of medications used to manage AF, such as propranolol and amiodarone (47, 48). Propofol is considered the gold standard for anesthetic induction, maintenance, and sedation during surgery in modern medicine. It prevents arrhythmias by significantly inhibiting atrioventricular nodal conduction and dose-dependently blocking cardiac ion channels, such as calcium and sodium channels (49). Additionally, propofol has a negative inotropic effect, primarily due to its blockade of L-type calcium channels, which reduces myocardial contractility (50). This effect leads to a shortening of the action potential duration and decreased myocardial contractility (51, 52). Furthermore, studies have shown that, during acute ischemia, propofol can reduce the variability of action potential duration between normal and ischemic regions, potentially mitigating spontaneous arrhythmias associated with myocardial reperfusion injury (53).

In summary, the mechanisms by which DEX and propofol prevent arrhythmias differ significantly. DEX primarily exerts its effects by modulating the balance of the autonomic nervous system, providing antioxidant and anti-inflammatory effects, and controlling vagal nerve activity. In contrast, propofol prevents arrhythmias through direct effects on cardiac electrophysiology and negative inotropic actions. These distinct mechanisms illustrate the different pathways through which DEX and propofol contribute to arrhythmia prevention.

### Clinical implication

Our study offers a comprehensive analysis of associated arrhythmia with DEX in comparison to the conventional drug propofol in cardiac surgery. Specifically, DEX's use substantially diminishes the risk of ventricular arrhythmias in patients. Furthermore, even mild bradycardia typically does not necessitate specific therapeutic intervention, underscoring its practical controllability.

### How to apply this knowledge?

The results of this study have important implications for guiding drug selection in adults after cardiac surgery. First of all, in cardiac surgery, it is recommended to include DEX in the anesthetic regimen to take advantage of its significant reduction in ventricular arrhythmias, which helps to improve postoperative cardiac stability and the overall prognosis of the patient. However, because DEX may increase the risk of bradycardia, strict monitoring measures must be implemented and the dosage of the drug adjusted according to the actual situation to reduce the occurrence of severe bradycardia. In high-risk patients with ventricular arrhythmias, such as those with coronary artery disease or heart failure, DEX may offer better outcomes than propofol by reducing arrhythmia risk. For individuals prone to bradycardia, including the elderly and those with conduction issues, propofol might be more beneficial. Thus, the choice between DEX and propofol should consider the specific arrhythmia risk of the patient. When considering the use of DEX as a potential treatment, physicians and clinical decision-makers can make more informed decisions by carefully evaluating the benefits of its anti-ventricular arrhythmic properties on the risk of bradycardia.

### What do we update?

While prior meta-analyses have explored the effects of DEX in comparison to placebo or mixed sedation drugs post-cardiac surgery (11, 29, 54). Both DEX and propofol are widely used as a sedative agent in operating rooms in cardiac surgery and ICUs. Our meta-analysis conducted a direct comparison between DEX and propofol on the effect of arrhythmias.

## Strengths and limitations

This is the first meta-analysis compared DEX and propofol, and evidence from RCTs indicates that DEX may be more clinically suitable than the commonly utilized propofol. However, it is important to acknowledge that there is a limited number of results per outcome, a small sample size, and potential heterogeneity that may affect the overall reliability of the results. In addition, the severity of bradycardia in the studies we included was not distinguished in detail, so we were unable to perform severity-based subgroup analyses to understand the specific effects of dexmedetomidine and propofol on different degrees of bradycardia. Additionally, the RCTs we included in our analysis lacked high-quality studies, further research is needed to expand the scope of the studies and gain a more comprehensive understanding of this association. Moreover, it's essential to highlight that our meta-analysis exclusively involves patients after cardiac surgery. Hence, additional research is warranted to ascertain the generalizability of these findings across various surgical procedures and to evaluate the enduring significance of their long-term effects.

## Conclusion

Our findings suggested that DEX exhibits stronger antiarrhythmic properties in in-hospital ventricular arrhythmias compared to propofol in adult patients undergoing cardiac surgery. However, the occurrence of in-hospital bradycardia with potential adverse effects should be taken into consideration when using DEX. Furthermore, while propofol has demonstrated effectiveness in terminating AF by previous findings, further research is warranted to establish whether DEX can effectively reduce the risk of AF when compared to propofol.

## Data Availability

The original contributions presented in the study are included in the article/Supplementary Material, further inquiries can be directed to the corresponding authors.
