# Supplementary material for: Dexmedetomidine vs. propofol on arrhythmia in cardiac surgery: a meta-analysis of randomized controlled trials
Source: Front Cardiovasc Med. 2024 Oct 10;11:1433841. doi: 10.3389/fcvm.2024.1433841 (PMC11499117; doi:10.3389/fcvm.2024.1433841)
Supplement: Supplementary file 1 [file Datasheet1.docx]

**Data Supplement**

Dexmedetomidine vs. propofol on arrhythmia in cardiac surgery: A meta-analysis with trial sequential analysis of randomized controlled trials

**Supplementary Table 1. PRISMA Checklist of included studies in this meta-analysis of Dexmedetomidine vs. Propofol effect on arrhythmia in cardiac surgery: a systematic review and meta-analysis of randomized controlled trials.**

| **Section/topic** | **#** | **Checklist item** | **Reported on page #** |
| --- | --- | --- | --- |
| **TITLE** | | |  |
| Title | 1 | Identify the report as a systematic review, meta-analysis, or both. |  |
| **ABSTRACT** | | |  |
| Structured summary | 2 | Provide a structured summary including, as applicable: background; objectives; data sources; study eligibility criteria, participants, and interventions; study appraisal and synthesis methods; results; limitations; conclusions and implications of key findings; systematic review registration number. |  |
| **INTRODUCTION** | | |  |
| Rationale | 3 | Describe the rationale for the review in the context of what is already known. |  |
| Objectives | 4 | Provide an explicit statement of questions being addressed with reference to participants, interventions, comparisons, outcomes, and study design (PICOS). |  |
| **METHODS** | | |  |
| Protocol and registration | 5 | Indicate if a review protocol exists, if and where it can be accessed (e.g., Web address), and, if available, provide registration information including registration number. |  |
| Eligibility criteria | 6 | Specify study characteristics (e.g., PICOS, length of follow-up) and report characteristics (e.g., years considered, language, publication status) used as criteria for eligibility, giving rationale. |  |
| Information sources | 7 | Describe all information sources (e.g., databases with dates of coverage, contact with study authors to identify additional studies) in the search and date last searched. |  |
| Search | 8 | Present full electronic search strategy for at least one database, including any limits used, such that it could be repeated. |  |
| Study selection | 9 | State the process for selecting studies (i.e., screening, eligibility, included in systematic review, and, if applicable, included in the meta-analysis). |  |
| Data collection process | 10 | Describe method of data extraction from reports (e.g., piloted forms, independently, in duplicate) and any processes for obtaining and confirming data from investigators. |  |
| Data items | 11 | List and define all variables for which data were sought (e.g., PICOS, funding sources) and any assumptions and simplifications made. |  |
| Risk of bias in individual studies | 12 | Describe methods used for assessing risk of bias of individual studies (including specification of whether this was done at the study or outcome level), and how this information is to be used in any data synthesis. |  |
| Summary measures | 13 | State the principal summary measures (e.g., risk ratio, difference in means). |  |
| Synthesis of results | 14 | Describe the methods of handling data and combining results of studies, if done, including measures of consistency (e.g., I^2^) for each meta-analysis. |  |
| Risk of bias across studies | 15 | Specify any assessment of risk of bias that may affect the cumulative evidence (e.g., publication bias, selective reporting within studies). |  |
| Additional analyses | 16 | Describe methods of additional analyses (e.g., sensitivity or subgroup analyses, meta-regression), if done, indicating which were pre-specified. |  |
| **RESULTS** | | |  |
| Study selection | 17 | Give numbers of studies screened, assessed for eligibility, and included in the review, with reasons for exclusions at each stage, ideally with a flow diagram. |  |
| Study characteristics | 18 | For each study, present characteristics for which data were extracted (e.g., study size, PICOS, follow-up period) and provide the citations. |  |
| Risk of bias within studies | 19 | Present data on risk of bias of each study and, if available, any outcome level assessment (see item 12). |  |
| Results of individual studies | 20 | For all outcomes considered (benefits or harms), present, for each study: (a) simple summary data for each intervention group (b) effect estimates and confidence intervals, ideally with a forest plot. |  |
| Synthesis of results | 21 | Present results of each meta-analysis done, including confidence intervals and measures of consistency. |  |
| Risk of bias across studies | 22 | Present results of any assessment of risk of bias across studies (see Item 15). |  |
| Additional analysis | 23 | Give results of additional analyses, if done (e.g., sensitivity or subgroup analyses, meta-regression [see Item 16]). |  |
| **DISCUSSION** | | |  |
| Summary of evidence | 24 | Summarize the main findings including the strength of evidence for each main outcome; consider their relevance to key groups (e.g., healthcare providers, users, and policy makers). |  |
| Limitations | 25 | Discuss limitations at study and outcome level (e.g., risk of bias), and at review-level (e.g., incomplete retrieval of identified research, reporting bias). |  |
| Conclusions | 26 | Provide a general interpretation of the results in the context of other evidence, and implications for future research. |  |
| **FUNDING** | | |  |
| Funding | 27 | Describe sources of funding for the systematic review and other support (e.g., supply of data); role of funders for the systematic review. |  |

*From:* Moher D, Liberati A, Tetzlaff J, Altman DG, The PRISMA Group (2009). Preferred Reporting Items for Systematic Reviews and Meta-Analyses: The PRISMA Statement. PLoS Med 6(7): e1000097. doi:10.1371/journal.pmed1000097

For more information, visit: **www.prisma-statement.org**.

**Supplementary Table 2. Specific search**

**PubMed database:**

| Search | Query |
| --- | --- |
| #1 | dexmedetomidine |
| #2 | Precedex |
| #3 | Dexmedetomidine Hydrochloride |
| #4 | Propofol |
| #5 | 2,6-Diisopropylphenol |
| #6 | 2,6-Bis(1-methylethyl)phenol |
| #7 | Cardiac Surgery |
| #8 | Heart Surgical Procedure |
| #9 | Arrythmia |
| #10 | Cardiac Dysrhythmia |
| #11 | #1 OR #2 OR #3 |
| #12 | #4 OR #5 OR #6 |
| #13 | #7 OR #8 |
| #14 | #9 OR #10 |
| #15 | #11 AND #12 AND #13 AND #14 |

**Embase database：**

| Search | Query |
| --- | --- |
| #1 | dexmedetomidine |
| #2 | Precedex |
| #3 | Dexmedetomidine Hydrochloride |
| #4 | Propofol |
| #5 | 2,6-Diisopropylphenol |
| #6 | 2,6-Bis(1-methylethyl)phenol |
| #7 | Cardiac Surgery |
| #8 | Heart Surgical Procedure |
| #9 | Arrythmia |
| #10 | Cardiac Dysrhythmia |
| #11 | #1 OR #2 OR #3 |
| #12 | #4 OR #5 OR #6 |
| #13 | #7 OR #8 |
| #14 | #9 OR #10 |
| #15 | #11 AND #12 AND #13 AND #14 |

**The Cochrane Library：**

| Search | Query |
| --- | --- |
| #1 | dexmedetomidine |
| #2 | Precedex |
| #3 | Dexmedetomidine Hydrochloride |
| #4 | Propofol |
| #5 | 2,6-Diisopropylphenol |
| #6 | 2,6-Bis(1-methylethyl)phenol |
| #7 | Cardiac Surgery |
| #8 | Heart Surgical Procedure |
| #9 | Arrythmia |
| #10 | Cardiac Dysrhythmia |
| #11 | #1 OR #2 OR #3 |
| #12 | #4 OR #5 OR #6 |
| #13 | #7 OR #8 |
| #14 | #9 OR #10 |
| #15 | #11 AND #12 AND #13 AND #14 |

**ClinicalTrails.gov**

| Search | Query |
| --- | --- |
| #1 | dexmedetomidine |
| #2 | Precedex |
| #3 | Dexmedetomidine Hydrochloride |
| #4 | Propofol |
| #5 | 2,6-Diisopropylphenol |
| #6 | 2,6-Bis(1-methylethyl)phenol |
| #7 | Cardiac Surgery |
| #8 | Heart Surgical Procedure |
| #9 | Arrythmia |
| #10 | Cardiac Dysrhythmia |
| #11 | #1 OR #2 OR #3 |
| #12 | #4 OR #5 OR #6 |
| #13 | #7 OR #8 |
| #14 | #9 OR #10 |
| #15 | #11 AND #12 AND #13 AND #14 |

**Supplementary Table 3. Studies excluded(n=18) with reasons**

| Studies excluded | Reasons (according to PICOS) |
| --- | --- |
| Maldonado et al-2009^1^ | No extractable related data |
| Zientara et al-2019^2^ | Retrospective study |
| Jakob et al-2012^3^ | Unrelated to the condition of heart surgery |
| Elgebaly et al-2020^4^ | No extractable related data |
| Sheikh et al-2018^5^ | No extractable related data |
| Elgebaly et al-2018^6^ | No extractable related data |
| Chang et al-2018^7^ | Unrelated to the condition of heart surgery |
| Mogahd et al-2018^8^ | No identical drug pairings |
| Soltani et al-2017^9^ | No extractable related data |
| Turan et al-2014^10^ | No identical drug pairings |
| Heybati et al- 2022^11^ | Unrelated to the condition of heart surgery |
| Abowali et al-2021^12^ | No extractable related data |
| Nishizawa et al-2017^13^ | Unrelated to the condition of heart surgery |
| Coursin et al-2001^14^ | No identical drug pairings |
| Zhu et al-2018^15^ | No identical drug pairings |
| Liu et al-2017^16^ | No identical drug pairings |
| Liu et al-2016^17^ | Data duplication |
| Homberg et al-2023^18^ | No extractable related data |

**Supplementary Figure 1. Quality assessment of the included studies by** **the Cochrane Risk of Bias 2.0(RoB2)**


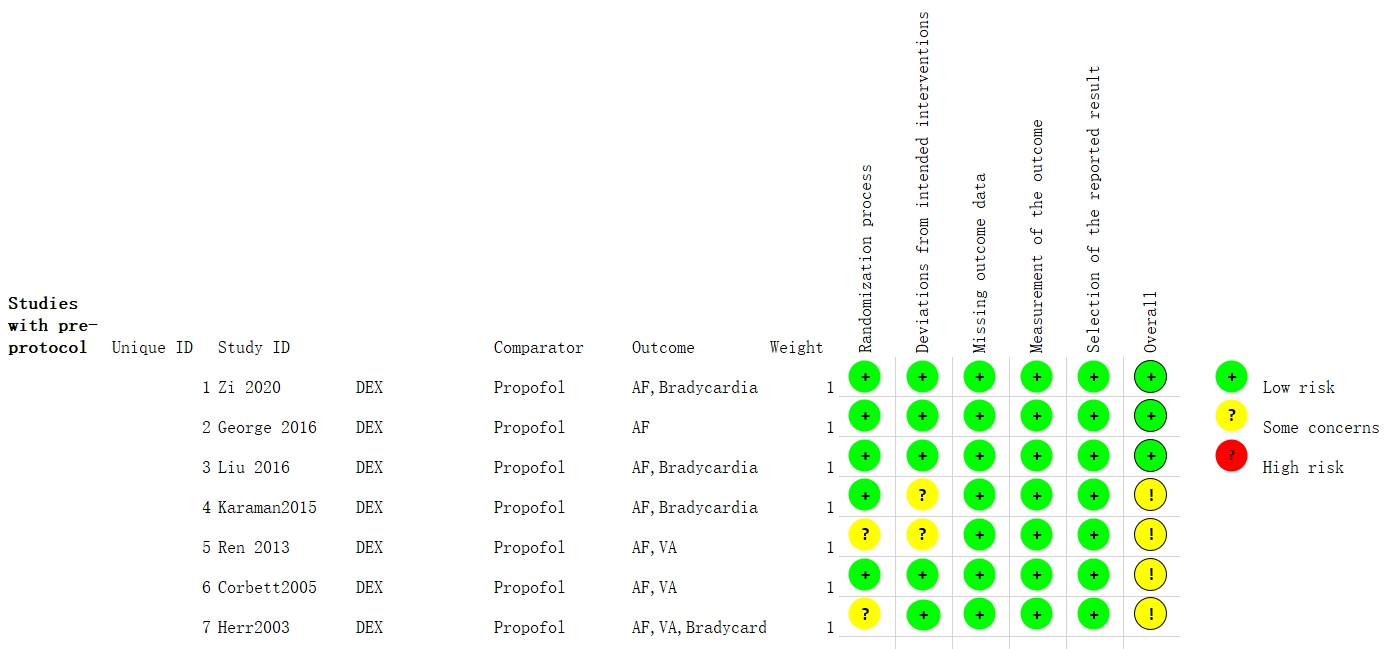


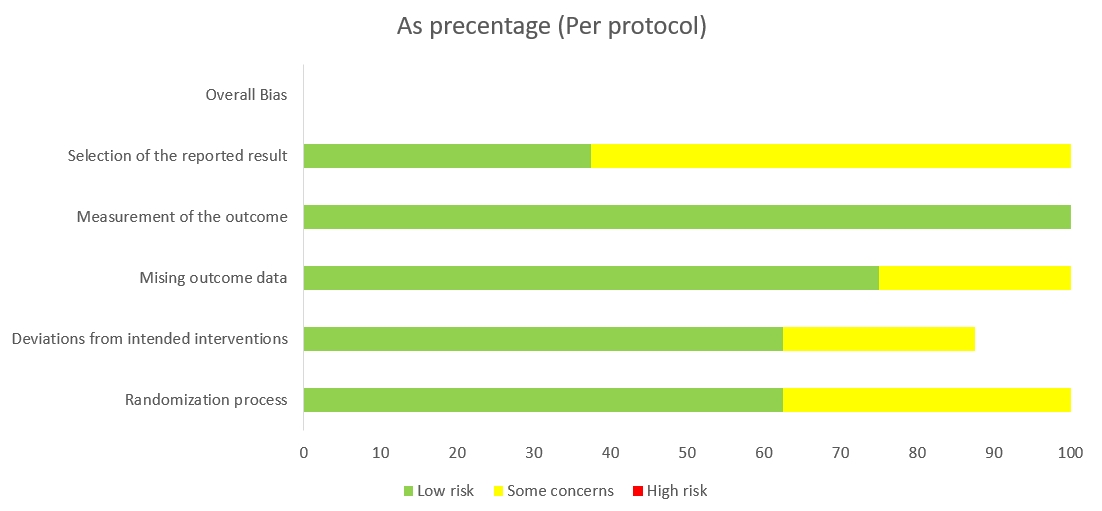


**Supplementary Table 4. GRADE evidence profile for the ventricular arrhythmias, bradycardia and atrial fibrillation.**

| **Quality assessment** | | | | | | | **No of patients** | | **Effect** | | **Quality** | **Importance** |  |
| --- | --- | --- | --- | --- | --- | --- | --- | --- | --- | --- | --- | --- | --- |
|  |  |  |  |  |  |  |  |  |  |  |  |  |  |
| **No of studies** | **Design** | **Risk of bias** | **Inconsistency** | **Indirectness** | **Imprecision** | **Other considerations** | **DEX** | **Control** | **Relative (95% CI)** | **Absolute** |  |  |  |
| **Bradycardia** | | | | | | | | | | | | |  |
| 4 | randomised trials | no serious risk of bias | no serious inconsistency | no serious indirectness | no serious imprecision | none | 14/285  (4.9%) | 5/285  (1.8%) | OR 2.88 (1.02 to 8.17) | 23 more per 1000 (from 6 fewer to 123 more) | ⊕⊕⊕⊕  HIGH | CRITICAL |  |
|  |  |  |  |  |  |  |  | 3.3% |  | 31 more per 1000 (from 9 fewer to 161 more) |  |  |  |
| **Ventricular Arrhythmias** | | | | | | | | | | | | |  |
| 3 | randomised trials | no serious risk of bias | no serious inconsistency | no serious indirectness | no serious imprecision | none | 1/272  (0.4%) | 14/274  (5.1%) | OR 0.14 (0.03 to 0.66) | 44 fewer per 1000 (from 17 fewer to 49 fewer) | ⊕⊕⊕⊕ HIGH | CRITICAL |  |
|  |  |  |  |  |  |  |  | 4.8% |  | 41 fewer per 1000 (from 16 fewer to 46 fewer) |  |  |  |
| **Atrial Fibrillation** | | | | | | | | | | | | |  |
| 7 | randomised trials | no serious risk of bias | serious^1^ | no serious indirectness | no serious imprecision | none | 85/500  (17%) | 102/504  (20.2%) | OR 0.69 (0.36 to 1.29) | 76 fewer per 1000 (from 144 fewer to 7 more) | ⊕⊕⊕O  MODERATE | CRITICAL |  |
|  |  |  |  |  |  |  |  | 18.6% |  | 52 fewer per 1000 (from 95 fewer to 5 more) |  |  |  |

**Figure S2. Funnel plots demonstrating potential publication bias in the included studies reporting Dexmedetomidine vs. Propofol effect on arrhythmia in cardiac surgery**

1. **B. C.**


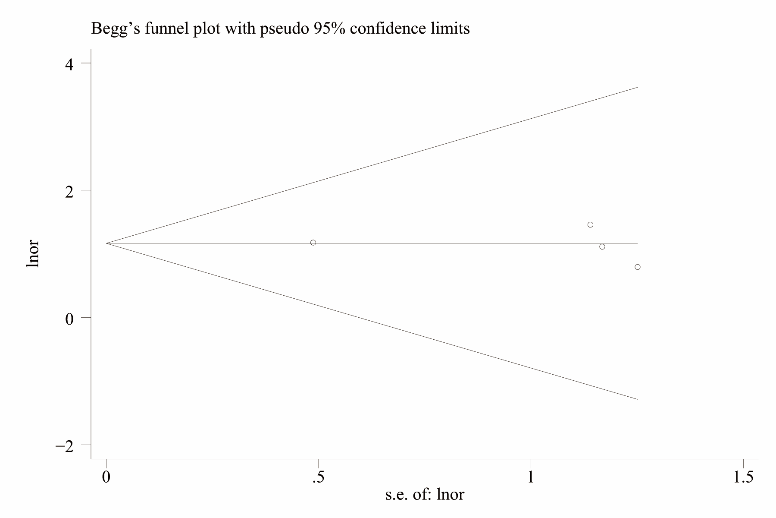

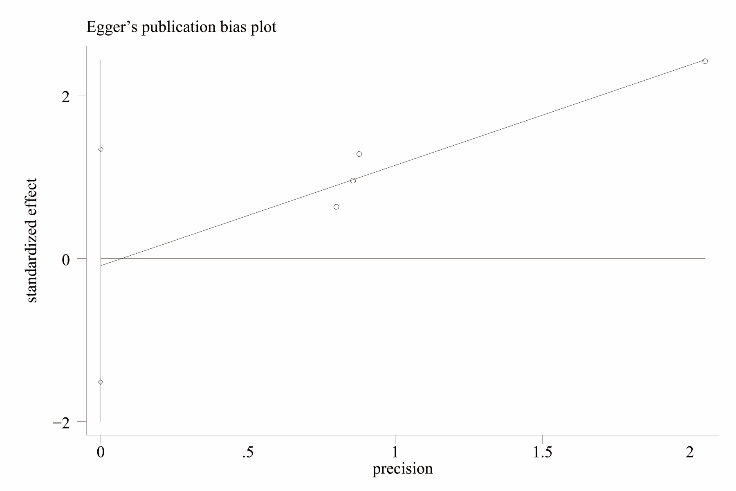

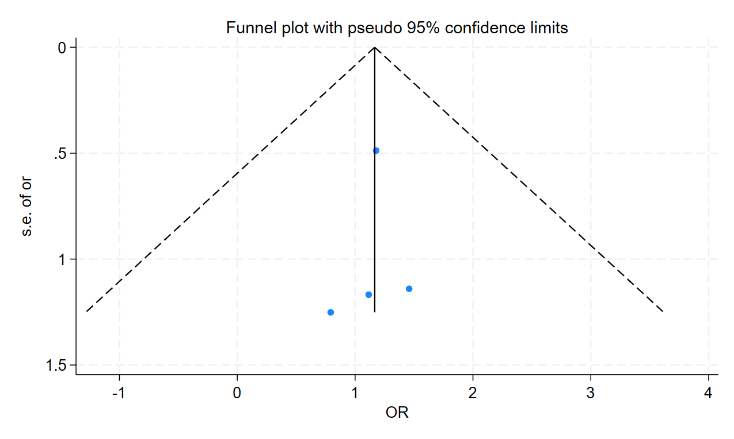


**
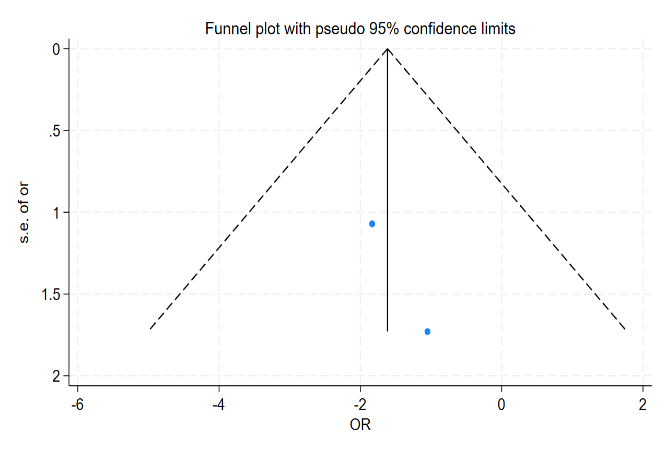

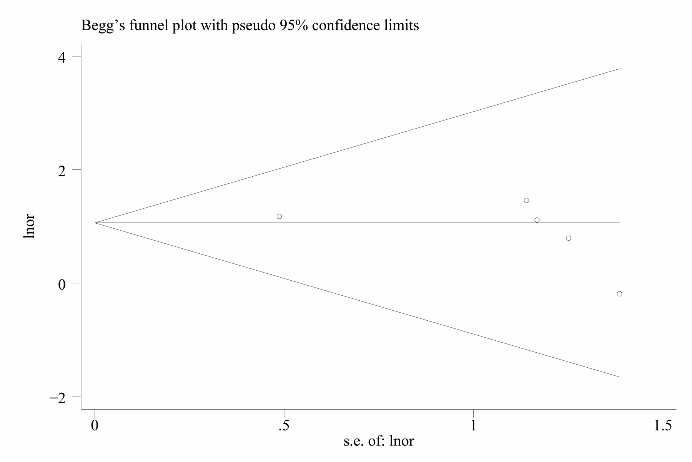

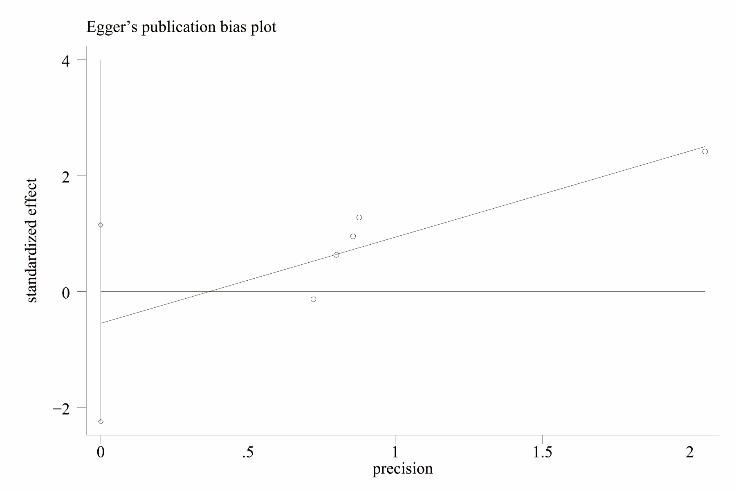
D. E. F.**

**
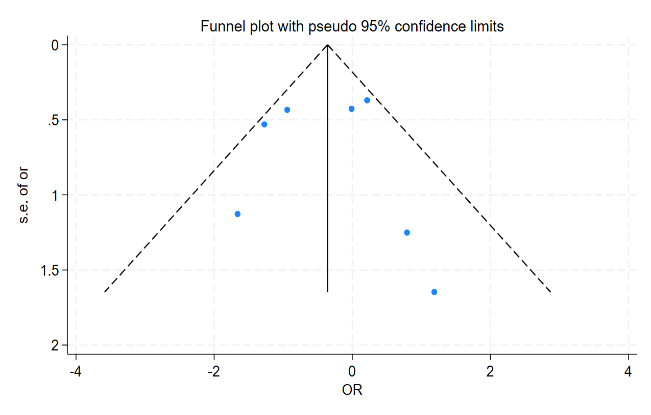

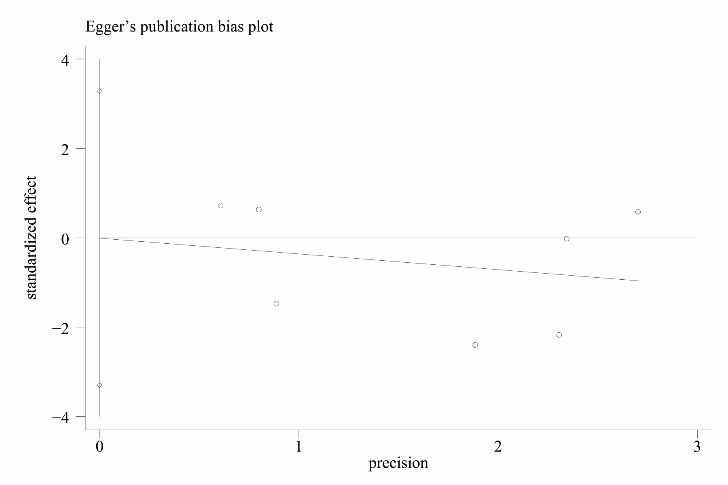

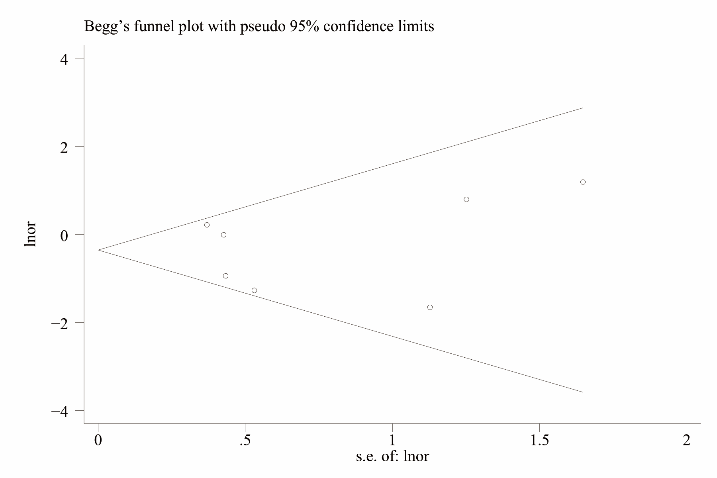
H. G. I.**

**Figure S3. Sensitivity analyses of included studies reporting Dexmedetomidine vs. Propofol effect on arrhythmia in cardiac surgery**

**A. B. C.**

**
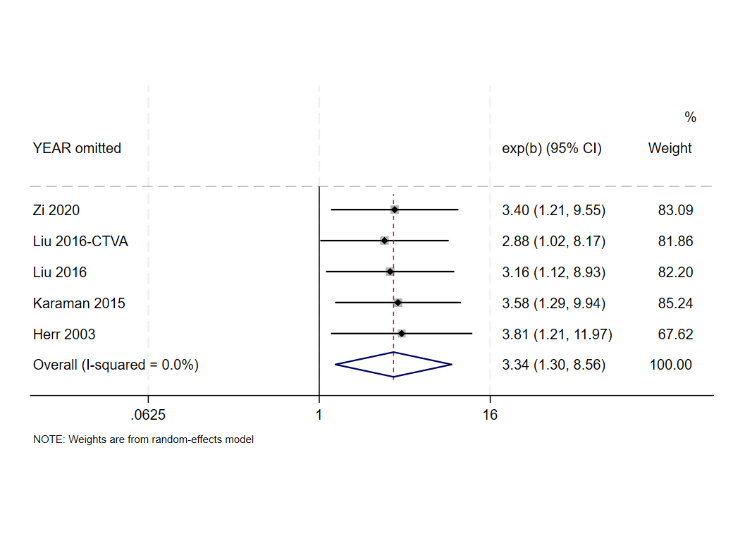

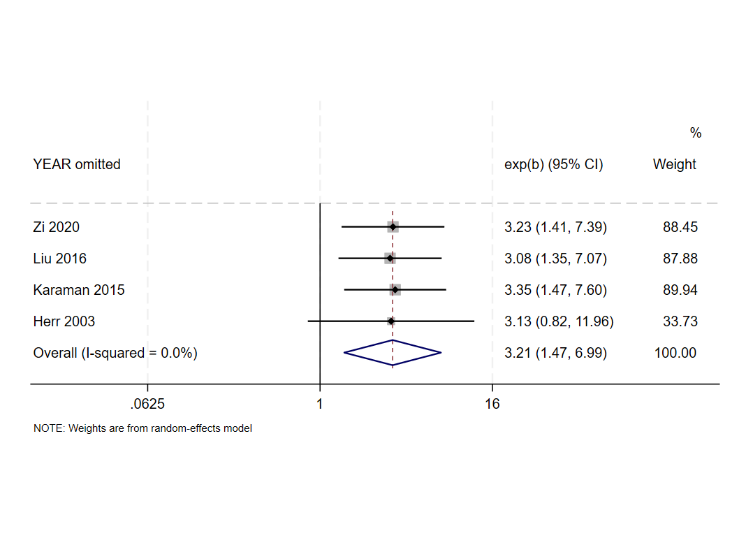

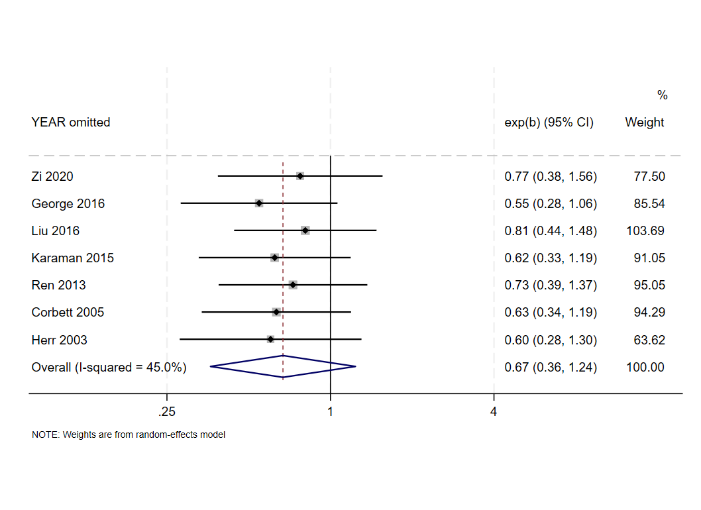
**

**Figure S4. The trial sequence analysis of included studies reporting Dexmedetomidine vs. Propofol effect on arrhythmia in cardiac surgery**

**
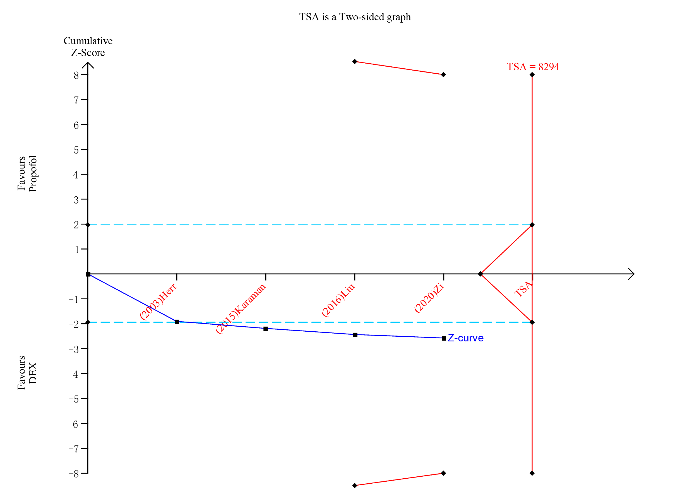

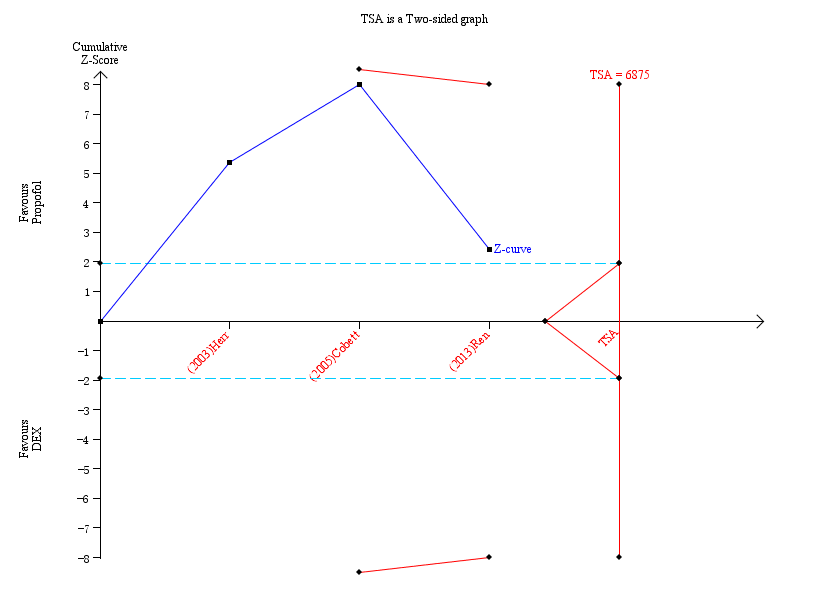

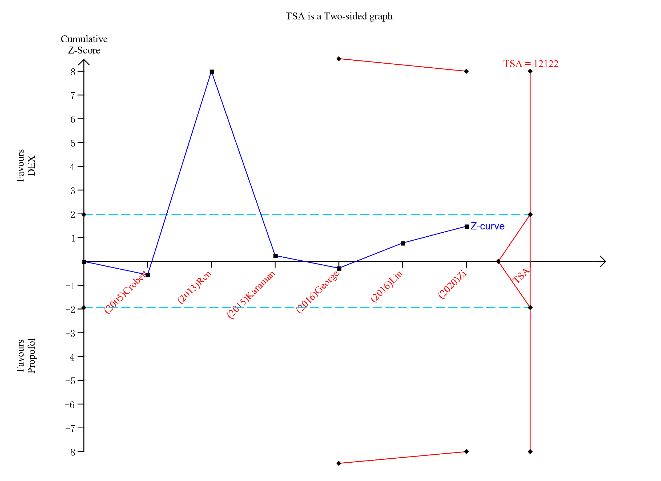
**

1. Maldonado JR, Wysong A, van der Starre PJ, Block T, Miller C, Reitz BA. Dexmedetomidine and the reduction of postoperative delirium after cardiac surgery. *Psychosomatics*. May-Jun 2009;50(3):206-17. doi:10.1176/appi.psy.50.3.206

2. Zientara A, Mariotti S, Matter-Ensner S, et al. Fast-Track Management in Off-Pump Coronary Artery Bypass Grafting: Dexmedetomidine Provides Rapid Extubation and Effective Pain Modulation. *The Thoracic and cardiovascular surgeon*. Sep 2019;67(6):450-457. doi:10.1055/s-0038-1668602

3. Jakob SM, Ruokonen E, Grounds RM, et al. Dexmedetomidine vs midazolam or propofol for sedation during prolonged mechanical ventilation: two randomized controlled trials. *Jama*. Mar 21 2012;307(11):1151-60. doi:10.1001/jama.2012.304

4. Elgebaly AS, Fathy SM, Sallam AA, Elbarbary Y. Cardioprotective effects of propofol-dexmedetomidine in open-heart surgery: A prospective double-blind study. *Annals of cardiac anaesthesia*. Apr-Jun 2020;23(2):134-141. doi:10.4103/aca.ACA_168_18

5. Sheikh TA, Dar BA, Akhter N, Ahmad N. A Comparative Study Evaluating Effects of Intravenous Sedation by Dexmedetomidine and Propofol on Patient Hemodynamics and Postoperative Outcomes in Cardiac Surgery. *Anesthesia, essays and researches*. Apr-Jun 2018;12(2):555-560. doi:10.4103/aer.AER_46_18

6. Elgebaly AS, Sabry M. Sedation effects by dexmedetomidine versus propofol in decreasing duration of mechanical ventilation after open heart surgery. *Annals of cardiac anaesthesia*. Jul-Sep 2018;21(3):235-242. doi:10.4103/aca.ACA_168_17

7. Chang YF, Chao A, Shih PY, et al. Comparison of dexmedetomidine versus propofol on hemodynamics in surgical critically ill patients. *The Journal of surgical research*. Aug 2018;228:194-200. doi:10.1016/j.jss.2018.03.040

8. Mogahd MM, Mahran MS, Elbaradi GF. Safety and efficacy of ketamine-dexmedetomidine versus ketamine-propofol combinations for sedation in patients after coronary artery bypass graft surgery. *Annals of cardiac anaesthesia*. Apr-Jun 2017;20(2):182-187. doi:10.4103/aca.ACA_254_16

9. Soltani G, Jahanbakhsh S, Tashnizi MA, et al. Effects of dexmedetomidine on heart arrhythmia prevention in off-pump coronary artery bypass surgery: A randomized clinical trial. *Electronic physician*. Oct 2017;9(10):5578-5587. doi:10.19082/5578

10. Turan A, Allen Bashour C, You J, et al. Dexmedetomidine sedation after cardiac surgery decreases atrial arrhythmias. Article. *Journal of clinical anesthesia*. 2014;26(8):634-642. doi:10.1016/j.jclinane.2014.05.009

11. Heybati K, Zhou F, Ali S, et al. Outcomes of dexmedetomidine versus propofol sedation in critically ill adults requiring mechanical ventilation: a systematic review and meta-analysis of randomised controlled trials. *British journal of anaesthesia*. Oct 2022;129(4):515-526. doi:10.1016/j.bja.2022.06.020

12. Abowali HA, Paganini M, Enten G, Elbadawi A, Camporesi EM. Critical Review and Meta-Analysis of Postoperative Sedation after Adult Cardiac Surgery: Dexmedetomidine Versus Propofol. *Journal of cardiothoracic and vascular anesthesia*. Apr 2021;35(4):1134-1142. doi:10.1053/j.jvca.2020.10.022

13. Nishizawa T, Suzuki H, Hosoe N, Ogata H, Kanai T, Yahagi N. Dexmedetomidine vs propofol for gastrointestinal endoscopy: A meta-analysis. *United European gastroenterology journal*. Nov 2017;5(7):1037-1045. doi:10.1177/2050640616688140

14. Coursin DB, Coursin DB, Maccioli GA. Dexmedetomidine. *Current opinion in critical care*. Aug 2001;7(4):221-6. doi:10.1097/00075198-200108000-00002

15. Zhu Z, Zhou H, Ni Y, Wu C, Zhang C, Ling X. Can dexmedetomidine reduce atrial fibrillation after cardiac surgery? A systematic review and meta-analysis. *Drug design, development and therapy*. 2018;12:521-531. doi:10.2147/dddt.S153834

16. Liu H, Ji F, Peng K, Applegate RL, 2nd, Fleming N. Sedation After Cardiac Surgery: Is One Drug Better Than Another? *Anesthesia and analgesia*. Apr 2017;124(4):1061-1070. doi:10.1213/ane.0000000000001588

17. Liu X, Zhang K, Wang W, et al. Dexmedetomidine Versus Propofol Sedation Improves Sublingual Microcirculation After Cardiac Surgery: A Randomized Controlled Trial. *Journal of cardiothoracic and vascular anesthesia*. Dec 2016;30(6):1509-1515. doi:10.1053/j.jvca.2016.05.038

18. Homberg MC, Bouman EAC, Joosten BAJ. Optimization of procedural sedation and analgesia during atrial fibrillation ablation. *Current opinion in anaesthesiology*. Jun 1 2023;36(3):354-360. doi:10.1097/aco.0000000000001263
